# Choroidal Metastasis From Papillary Renal Cell Carcinomas: A Case Report and a Review of the Literature

**DOI:** 10.7759/cureus.60191

**Published:** 2024-05-13

**Authors:** Shinnosuke Hiruta, Go Kaneko, Yu Miyama, Yousuke Miyasaka, Yuta Umezawa, Masayuki Hagiwara, Suguru Shirotake, Kent Kanao, Masanori Yasuda, Masafumi Oyama

**Affiliations:** 1 Urologic Oncology, Saitama Medical University International Medical Center, Hidaka, JPN; 2 Pathology, Saitama Medical University International Medical Center, Hidaka, JPN; 3 Ophthalmology, Saitama Medical University International Medical Center, Hidaka, JPN

**Keywords:** immune checkpoint inhibitor, renal cell carcinoma, non-clear cell renal carcinoma, kidney papillary renal cell carcinoma, molecular-targeted therapy, stereotactic radiotherapy, choroidal metastasis

## Abstract

Choroidal metastasis originating from renal cell carcinomas (RCCs) is rare. To the best of our knowledge, 31 cases of choroidal metastasis from RCC have been reported in the English literature as of January 31, 2024. Nevertheless, physicians need to be vigilant in recognizing this condition, as its progression impacts the quality of life (QOL) of affected patients. In Case 1, a 60-year-old male with a medical history of papillary RCC experienced a deterioration in visual acuity (VA) and was diagnosed with solitary choroidal metastasis. Subsequently, multiple metastases were identified, prompting the initiation of a combination therapy regimen consisting of pembrolizumab plus axitinib. Despite treatment, progression of choroidal metastasis and a further decline in VA were observed. The patient underwent stereotactic radiotherapy and experienced complete resolution of the choroidal metastasis, accompanied by a slight improvement in VA. In Case 2, a 76-year-old man presented with a renal tumor accompanied by lung metastases. He underwent nephrectomy, and the histological diagnosis was papillary RCC. We initiated combination therapy consisting of nivolumab plus cabozantinib. The patient experienced a decrease in VA during treatment. We identified extensive fine metastases scattered throughout the bilateral choroid. We administered axitinib, but the patient experienced bilateral blindness. Given the absence of established therapy for choroidal metastasis, it is crucial to maintain flexibility in treatment selection. Local or systemic approaches should be used as deemed appropriate for each individual case.

## Introduction

The choroid is the most common ocular structure affected by metastasis [1]. In a review of 520 patients presenting with 950 uveal metastases, the choroid was implicated in 88% of cases [1]. The most common primary sources of choroidal metastasis are breast cancer (40-53%) and lung cancer (20-29%) [1,2]. Less common primary sites include the gastrointestinal tract (4%), prostate (2%), kidney (2-4%), and skin (2%) [1-3]. The decision-making process for treating choroidal metastasis should flexibly consider various factors, such as the patient’s systemic status and the number and location of metastases. Early intervention is necessary due to the potential for progression leading to blindness and significant deterioration in the quality of life (QOL).

Herein, we present two cases of choroidal metastasis from papillary renal cell carcinoma (RCC), which were treated with radiation or medical treatment, including combination therapy consisting of immune checkpoint inhibitor and molecular targeted therapy.

## Case presentation

Case 1

**Figure 1:**
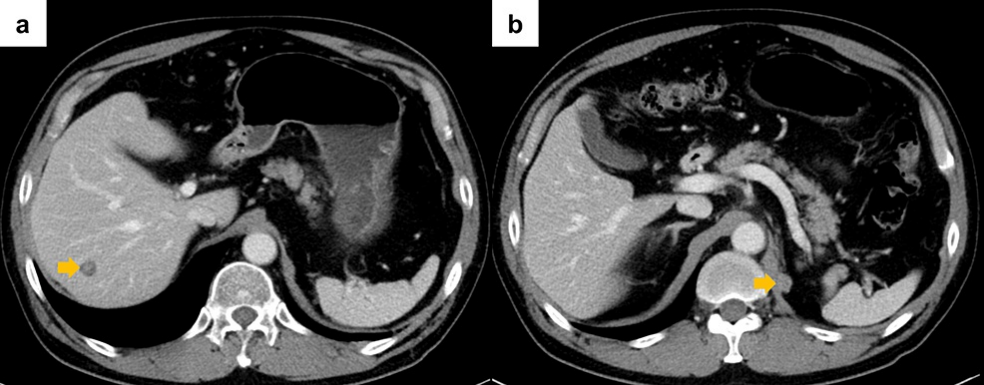
Abdominal computed tomography a) Liver metastasis and b) metastasis to the crus of the diaphragm

**Figure 2:**
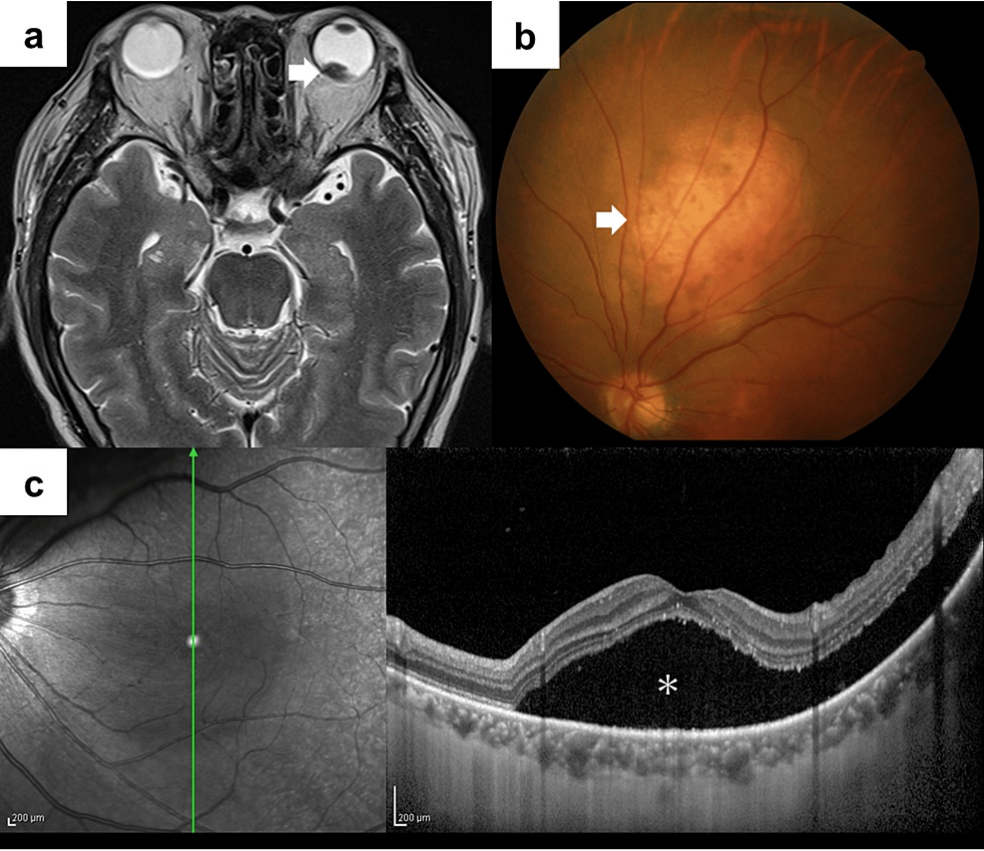
Magnetic resonance imaging, fundoscopy, and optical coherence tomography of the choroidal metastasis a: Magnetic resonance imaging (T2-weighted imaging) revealing an intraocular tumor in the left eye (arrow). b: Fundoscopy demonstrates pigmentary changes suggesting a neoplastic lesion (arrow). c: Optical coherence tomography at the level of the green arrow shows subretinal fluid and serous retinal detachment (*).

**Figure 3:**
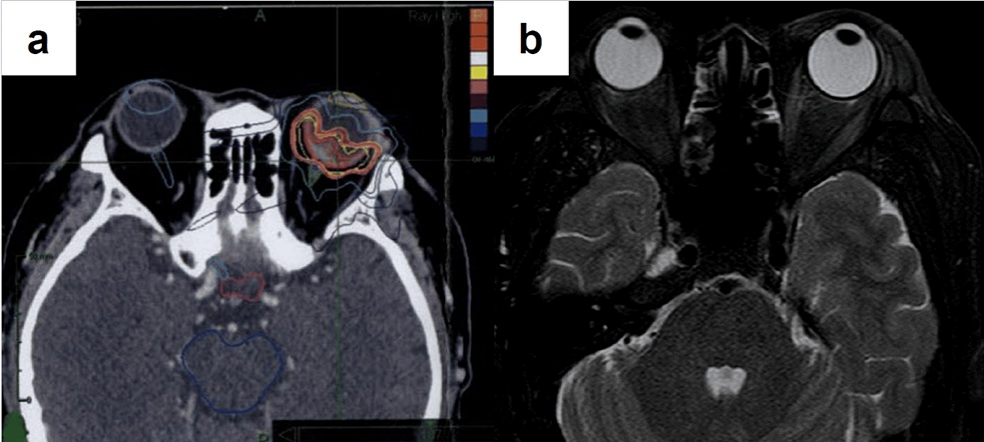
Stereotactic radiotherapy for the choroidal metastasis a: Planning computed tomography for stereotactic radiotherapy using CyberKnife for the choroidal metastasis in the left eye. b: Magnetic resonance imaging (fat-suppressed T2-weighted image) taken 1.2 months after irradiation shows that the choroidal metastasis in the left eye had disappeared entirely.

A 60-year-old Japanese male underwent a left partial nephrectomy at a neighboring hospital seven years earlier and he was diagnosed with RCC (pT1a). Three years later, he underwent a left radical nephrectomy due to a recurrence in the same kidney. The diagnosis was papillary RCC (WHO/ISUP Grade 3, pT3a). He began experiencing a decline in vision in his left eye about one year before presentation. Magnetic resonance imaging (MRI) at the ophthalmology department of a nearby hospital showed suspected choroidal metastasis, prompting a scheduled short-term follow-up. Subsequently, computed tomography (CT) scans conducted every six months showed metastases in the liver and left retroperitoneal space (Figure 1a, 1b). He was referred to our hospital. We initiated a combination regimen consisting of pembrolizumab (200 mg, every three weeks) plus axitinib (5 mg, twice daily) as first-line therapy. There was a slight reduction in the size of the metastatic lesions at 1.9 months after initiating therapy. However, an immune-related adverse event manifested, characterized by elevated transaminase elevation (aspartate aminotransferase: 379 U/L, alanine aminotransferase: 323 U/L). The first-line therapy was discontinued, and the patient was switched to systemic steroid therapy. The visual acuity in his left eye decreased from 20/25 to 20/300 at 3.6 months after the initiation of first-line therapy, and an increase in intraocular tumor size was observed on MRI (Figure 2a). Fundoscopy demonstrated pigmentary changes suggesting a neoplastic lesion in his left eye (Figure 2b). Optical coherence tomography revealed subretinal fluid and serous retinal detachment (Figure 2c). Stereotactic radiotherapy (SRT) using CyberKnife with a dose of 31 Gy delivered in five fractions was performed for the choroidal metastasis (Figure 3a). At 1.2 months after initiation of irradiation, MRI revealed the complete disappearance of the choroidal metastasis (Figure 3b). However, metastases in the liver and the retroperitoneal space worsened, leading to subsequent therapy using cabozantinib (second line) and nivolumab (third line). There was no recurrence of choroid involvement, and visual acuity recovered to 20/63 at 2.6 months after radiotherapy.

Case 2

**Figure 4:**
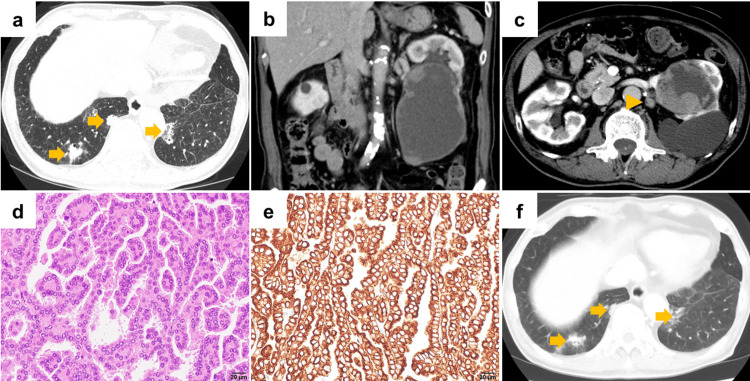
Computed tomography and microscopic findings a: Chest computed tomography reveals multiple lung metastases in the bilateral lower lobes (arrow). b: Abdominal computed tomography reveals a left renal cystic tumor measuring 10.7 cm with thickened irregular walls and solid components enhanced with contrast medium. c: Lymph node enlargement identified in the left renal hilum (arrow). d: Hematoxylin and eosin staining of the kidney tumor. Tumor cells form a papillary architecture. The scale bar in the lower right corner indicates 20 μm. e: Immunohistochemistry for cytokeratin 7 of the kidney tumor. Tumor cells are positive for cytokeratin 7. The scale bar in the lower right corner indicates 20 μm. f: Multiple lung metastases slightly decrease in size with combination therapy consisting of nivolumab and cabozantinib.

**Figure 5:**
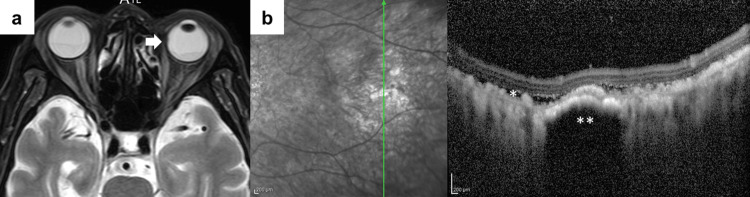
Magnetic resonance imaging and optical coherence tomography of choroidal metastases a: Magnetic resonance imaging (fat-suppressed T2-weighted image) suggesting the presence of a left intraocular tumor (arrows). b: Optical coherence tomography at the level of the green arrow reveals serous retinal detachment (*) and extensive fine metastases scattered throughout the choroid (**).

A 76-year-old Japanese male with a history of hypertension, hyperuricemia, atrial fibrillation, and hypertrophic cardiomyopathy visited a nearby hospital with a complaint of back discomfort. Chest CT showed bilateral lung masses. He was referred to our hospital's Department of Respiratory Medicine for further examination. Chest CT revealed multiple lung nodules, ranging from 0.5 cm to 2.5 cm, in both lower lobes (Figure 4a). Abdominal CT imaging unveiled a left cystic renal tumor (10.7 cm in diameter) exhibiting thickened irregular walls and solid components enhanced with contrast medium, alongside lymph node enlargement at the renal hilum (Figure 4b, 4c). The cystic renal tumor corresponded to category Ⅳ according to the Bosniak classification, and RCC with lymph node metastasis and multiple lung metastases (cT2bN1M1) was suspected. A bronchoscopic lung tumor biopsy was performed for histopathological diagnosis. Non-clear cell RCC was suspected; however, a definitive diagnosis could not be established due to insufficient specimens. He underwent laparoscopic nephrectomy and lymphadenectomy for definitive histopathological diagnosis and cytoreduction. The diagnosis was papillary RCC (WHO/ISUP Grade 2, pT3aN1) (Figure 4d, 4e), and the resected margin was negative. Combination therapy consisting of nivolumab (240 mg, every two weeks) plus cabozantinib (20 mg, once daily) was administered as first-line therapy. Cabozantinib was initiated at a reduced dose due to decreased cardiac function. Multiple lung metastases slightly decreased in size at 3.2 months after initiating combination therapy (Figure 4f). However, the patient noticed a decreased visual acuity in both eyes (right: from 20/20 to 20/125, left: from 20/25 to 20/63) and visited the Department of Ophthalmology. MRI demonstrated left intraocular tumors (Figure 5a). Optical coherence tomography revealed extensive fine tumors scattered throughout the choroid (Figure 5b), with similar findings noted bilaterally. Based on these findings, multiple choroidal metastases of RCC were suspected. Radiation therapy was initially considered; however, it was deemed inappropriate due to the potential for radiation-related complications arising from extensive exposure to both eyes. Consequently, axitinib (5 mg, twice daily) was initiated as a second-line therapy. The best response was defined as stable disease (Response Evaluation Criteria in Solid Tumors 1.1); however, we observed progression in both the size and number of choroidal metastases, resulting in bilateral blindness.

## Discussion

We described two cases involving radiation or medical therapy for choroidal metastasis from papillary RCC. One case consisted of solitary metastasis treated with SRT. In this case, the tumor resolved without adverse events, and visual acuity improved. In the other case, extensive fine metastases were scattered throughout the bilateral choroid. Medical therapy proved ineffective, resulting in the patient experiencing bilateral blindness. 

Choroidal metastasis treatment depends on various factors [4]. Observation is the preferred approach for patients with poor performance status. Medical therapies, such as chemotherapy, immunotherapy, hormone therapy, or whole-eye radiotherapy, are chosen when metastases are multifocal or bilateral. Immunotherapy has recently become the mainstream medical therapy for several cancers, including RCC. Its efficacy for choroidal metastasis has been reported [5,6]. On the other hand, SRT, plaque radiotherapy, transpupillary radiotherapy, or photodynamic therapy are selected for solitary metastasis. Enucleation is chosen for blind, painful eyes [4].

SRT has been widely used to treat intraocular tumors because it reduces tumor margins and preserves critical normal structures around the tumor, such as the optic nerve, lens, and lacrimal gland [7]. Recent studies have highlighted the favorable outcomes of SRT for choroidal metastases originating from various cancers [7-10]. Bellmann et al. reported the efficacy of SRT in the treatment of unifocal choroidal metastases, encompassing cases of breast cancer (three cases), lung cancer (three cases), colon cancer (three cases), and cutaneous melanoma (one case) [7]. SRT was administered with a total dose of 40 Gy delivered in five fractions, resulting in successful local tumor control across all patients over a median follow-up period of 6.5 months. In the present study, SRT demonstrated high effectiveness without adverse events in the treatment of Case 1. SRT may need to be actively chosen in solitary metastasis cases due to its high efficacy and lower incidence of adverse events; however, the appropriate irradiation dose and schedule have not been established.

To the best of our knowledge, 31 cases of choroidal metastasis from RCC have been reported in the English literature as of January 31, 2024. Histological types of RCC were described in only nine cases, with clear cell RCC and tubulopapillary RCC accounting for 88.9% and 11.1%, respectively. Local treatment for choroidal metastasis consisting of extra beam radiation therapy (five cases, 35.7%), plaque radiotherapy (two cases, 14.3%), mass resection (two cases, 14.3%), and enucleation (five cases, 35.7%) was performed. The outcomes of choroidal metastasis were reported in four out of seven cases treated with radiation therapy, demonstrating a high efficacy rate (75.0%). Among medical therapies for RCC, molecular targeted therapy, notably vascular endothelial growth factor inhibitors, and immunotherapy have emerged as mainstream treatments. A case study reported the regression of choroidal metastasis following treatment with sunitinib in a patient with multiple metastases that had not undergone local treatment [11]. Regarding choroidal melanoma, a study reported the efficacy of combination immunotherapy utilizing ipilimumab and nivolumab [5,12], suggesting that immunotherapy may be effective for the management of choroidal metastasis originating from RCC. Although a case involving multiple metastases treated with nivolumab has been reported [13], the specific outcome concerning choroidal metastasis was not described. In the present study, Case 2 was administered a combination therapy, consisting of an immune checkpoint inhibitor and molecular targeted therapy, for bilateral choroidal metastases; however, it proved to be ineffective.

## Conclusions

Choroidal metastasis originating from RCC is rare; nevertheless, physicians must be vigilant in identifying this condition as its progression can substantially degrade patient QOL. Early interventions should be prioritized to enhance the prognosis of ocular symptoms. Given the absence of a standardized therapy for choroidal metastasis stemming from RCC, it is important to adopt a flexible approach in selecting between local or systemic treatments.

## References

[1] Shields Shields, C.L. C.L., J.A. Shields, N.E. Gross, G.P. Schwartz, and S.E (1997). Lally: Survey of 520 eyes with uveal metastases. Ophthalmology.

[2] Ferry AP, Font RL (1974). Carcinoma metastatic to the eye and orbit. I. A clinicopathologic study of 227 cases. Arch Ophthalmol.

[3] Harbour Harbour, J.W. J.W., P. De Potter, C.L. Shields, and J.A (1994). Shields: Uveal metastasis from carcinoid tumor. Clinical observations in nine cases. Ophthalmology.

[4] Arepalli S, Kaliki S, Shields CL (2015). Choroidal metastases: origin, features, and therapy. Indian J Ophthalmol.

[5] Morkos M, Jain P, Pavlick AC, Finger PT (2020). Ipsilateral metastatic choroidal melanoma responds to systemic immunotherapy. Eur J Ophthalmol.

[6] Barrett D, Sumnicht A, Chalam KV, Rauser M (2021). Pembrolizumab dramatically resolves choroidal metastatis from esophageal adenocarcinoma and restores vision: a case report. Oxf Med Case Reports.

[7] Bellmann C, Fuss M, Holz FG (2000). Stereotactic radiation therapy for malignant choroidal tumors: preliminary, short-term results. Ophthalmology.

[8] Shimojima Y, Hirose Y, Nomura T (2023). Solitary choroidal metastasis of distal cholangiocarcinoma: a case report. Oncol Lett.

[9] Oliverio GW, Tedesco GR, Azzaro C, Meduri A, Aragona P (2022). Coexisting choroidal and brain metastases in a patient with breast cancer treated with stereotactic radiotherapy. Case Rep Ophthalmol.

[10] Haidar Y, Korn B, Rose M (2013). Complete regression of a choroidal metastasis secondary to breast cancer with stereotactic radiation: case report and review of literature. J Radiosurg SBRT.

[11] Chin EK, Almeida DR, Sacher BA, Boldt HC (2015). Rapid involution of choroidal metastasis secondary to renal cell carcinoma with oral sunitinib. JAMA Ophthalmol.

[12] Duong RT, Ambati NR, Peddada KV, Elghawy O, Gaughan EM, Shildkrot Y (2022). Multiple evanescent white dot syndrome-like reaction associated with ipilimumab and nivolumab immune checkpoint inhibitor therapy for metastasis of choroidal melanoma. Am J Ophthalmol Case Rep.

[13] Georgakopoulos CD, Pallikari A, Plotas P, Makri OE (2020). Late-onset bilateral choroidal metastases from clear cell renal cell carcinoma. Case Rep Urol.

